# Effects of Colonization, Geography and Environment on Genetic Divergence in the Intermediate Leaf-Nosed Bat, *Hipposideros larvatus*

**DOI:** 10.3390/ani11030733

**Published:** 2021-03-08

**Authors:** Xiangfeng Meng, Tong Liu, Lin Zhang, Longru Jin, Keping Sun, Jiang Feng

**Affiliations:** 1 Jilin Provincial Key Laboratory of Animal Resource Conservation and Utilization, Northeast Normal University, Changchun 130117, China; mengxf940@nenu.edu.cn (X.M.); liut035@nenu.edu.cn (T.L.); zhangl309@nenu.edu.cn (L.Z.); fengj@nenu.edu.cn (J.F.); 2 Key Laboratory of Vegetation Ecology, Ministry of Education, Changchun 130024, China; 3 School of Life Science, Jilin Agricultural University, Changchun 130118, China

**Keywords:** bat, genetic differentiation, evolutionary force, island

## Abstract

**Simple Summary:**

It is vitally important to unravel the evolutionary processes and potential drivers of genetic differentiation among populations in evolutionary biology. However, it remains unexplored how those drivers act on the genetic divergence among bat populations. In this study, we studied the genetic structure and evolutionary history of a bat species, *Hipposideros larvatus* from mainland China and Hainan Island. Using microsatellites and mtDNA to analyze genetic differentiation, we found an obvious genetic structure between the mainland and the island in *H. larvatus*. Integrating the genetic data, geography and climatic factors, we uncovered the combined effects including geography, environment and colonization history, on the genetic variation in *H. larvatus*. Our results are valuable in understanding the complex evolutionary processes among bat populations and provide implications for the conservation of island bat populations.

**Abstract:**

Determining the evolutionary history and population drivers, such as past large-scale climatic oscillations, stochastic processes and ecological adaptations, represents one of the aims of evolutionary biology. *Hipposideros larvatus* is a common bat species in Southern China, including Hainan Island. We examined genetic variation in *H. larvatus* using mitochondrial DNA and nuclear microsatellites. We found a population structure on both markers with a geographic pattern that corresponds well with the structure on mainland China and Hainan Island. To understand the contributions of geography, the environment and colonization history to the observed population structure, we tested isolation by distance (IBD), isolation by adaptation (IBA) and isolation by colonization (IBC) using serial Mantel tests and RDA analysis. The results showed significant impacts of IBD, IBA and IBC on neutral genetic variation, suggesting that genetic variation in *H. larvatus* is greatly affected by neutral processes, environmental adaptation and colonization history. This study enriches our understanding of the complex evolutionary forces that shape the distribution of genetic variation in bats.

## 1. Introduction

Uncovering the evolutionary processes that drive genetic differentiation among populations is a central goal of evolutionary biology [1,2]. Neutral processes like random genetic drift can drive the genetic divergence of populations. In this scenario, genetic differentiation at neutral markers is typically driven by isolation by distance (IBD), which is an increase in genetic differentiation associated with geographic distance between populations [3,4]. This suggests that neutral and stochastic processes can drive genetic differentiation among populations.

Selective or adaptive processes can also drive genetic divergence among populations. Selection can be involved in driving differentiation at neutral DNA markers [5,6]. In cases where populations exist in heterogeneous environments, individuals are likely to be adapted to local conditions. Gene flow among populations may be restricted by adaptive processes, resulting in isolation by adaptation (IBA) and prediction of a relationship between genetic distance and environmental distance [7]. IBA received both theoretical and empirical support and may be more common than previously realized [1]. Several studies distinguished IBA from IBD using neutral genetic markers [8,9,10].

Historic events, such as glacial vicariance or founder events, are additional factors that can affect population differentiation, which is termed “isolation by colonization (IBC)” [1,5,11]. However, it is difficult to distinguish IBC patterns generated by colonization history from IBD and IBA, because they might produce similar genetic differentiation. For example, local adaptation can reinforce founder effects and result in patterns similar to IBA [2]. Postglacial recolonization can also generate allele frequency gradients similar to IBA or IBD when colonization routes covary with environmental clines [12,13].

The importance of geography, the environment and colonization history in driving genetic divergence is of great interest [1,5,14,15]. However, the drivers, including geography, landscape features and colonization history, on population structure might not be mutually exclusive, but are more likely to be combined for natural populations [1]. 

Bats (Chiroptera) are the second largest order of mammals (ca. 1400 species). They are distributed globally [16] and are important biodiversity indicator species. This makes bats potential target organisms for evolutionary studies. However, in contrast to the rich biodiversity of bats, studies on their genetic structure and drivers of genetic divergence are limited [17,18]. 

*Hipposideros larvatus*, the intermediate leaf-nosed bat, is widely distributed in Asia, including China, Bangladesh, Indonesia, Malaysia, Myanmar and Northeast India [19]. This species is a good study system for testing the effects of IBD, IBA and IBC on genetic differentiation among populations. In China, *H. larvatus* is widespread and occurs in heterogeneous environments, even in different climatic zones across the south of mainland China and Hainan Island [20]. For example, a tropical monsoon climate exists on Hainan Island, but a subtropical monsoon climate exists on the adjacent mainland (e.g., Guangxi and Guangdong Provinces). This suggests that isolation by adaptation might contribute to the existing genetic structure and drive genetic differentiation between island and continental populations. Hainan Island is the second largest island in China and is separated from the mainland by the Qiongzhou Strait, which is 30 km wide on average. The relatively short distance of the strait suggests that it is not a major obstacle to animal movement between the mainland and the island. However, genetic differentiation was detected for some species distributed in mainland China and on Hainan Island. These species include the leaf-nosed bat (*Hipposideros armiger*) [21], the Asian honey bee (*Apis cerana*) [22] and the evergreen oak (*Quercus championii*) [23]. Thus, the effects of this historic vicariance need to be studied. To better understand the evolution of genetic structure in *H. larvatus*, it is necessary to combine multiple factors to reveal genetic differentiation.

Our research goals were to (i) describe levels of genetic diversity and the current genetic structure in *H. larvatus*, (ii) reconstruct the recent demographic history of this species and (iii) test for correspondence between genetic differentiation, the environment, geography and colonization history. To do this, we sampled populations across the range of *H. larvatus* in mainland China and Hainan Island and examined genetic variation in both nuclear microsatellites and mitochondrial (mt) DNA. We tested for IBD and IBA by comparing two neutral genetic markers with geographic and climate differences among the sampled populations. To disentangle these effects from IBC, maternal mitochondrial markers were incorporated to describe the historic genetic signatures and colonization history [15]. Colonization history can persist in mtDNA for a longer time due to the philopatry of female bats and their low migration. We used multiple methods to examine the relative roles of geographic and adaptive isolation as well as colonization history in determining the genetic variation of *H. larvatus*.

## 2. Material and Methods

### Sampling Collection

**Figure 1:**
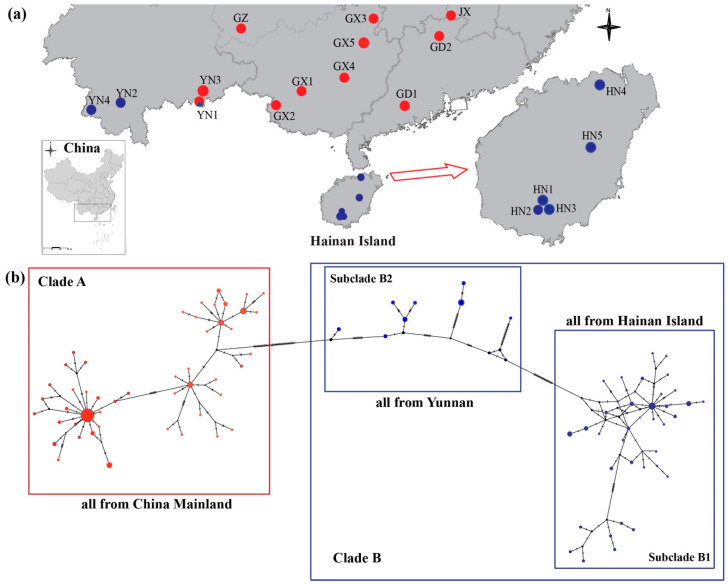
Sampling sites (**a**) and mitochondrial DNA nested haplotype clade (**b**) for *Hipposideros larvatus* in China.

Samples were collected from 18 populations of *H. larvatus* across the continent and Hainan Island in China (Figure 1a, Table 1). We used mist nets to capture bats at the entrance of the caves at night. After species identification, 3 mm wing biopsies were collected from 165 adult individuals and were preserved in 99% ethanol for DNA extraction. After sampling, all of the bats were released in their habitats. All field studies were approved by the Animal Research Authority in Northeast Normal University, China (approval number: NENU-20080416).

For each sampling site, we determined the latitude and longitude using GPS (eTrex Vista) and downloaded 19 bioclimatic variables, including a variety of temperature and precipitation data from CHELSA (resolution: 30 arcsec, ~1 km; time period: 1979–2013) [24,25] by the R package “raster” v.3.0-12 [26] (Supporting Information Table S1), to determine the environments experienced by each population. To identify the most informative, but least correlated, variables from the climate dataset, a principal component analysis (PCA) was conducted on the standardized environmental variables in R package “vegan” v.2.5-6 [27] to avoid potential collinearity among multiple climatic factors. The first three principal components (PCs), which accounted for 54.62%, 20.93% and 13.79% (Supporting Information Figure S1) of the variation, were used to compare the genetic differences among populations.

## 3. Genetic Data Collection

### 3.1. DNA Extraction and Sequencing

Total genomic DNA was extracted from bat wing membranes by a UNIQ-10 column animal genomic DNA isolation kit (Sangon, China). For each individual, the complete cytochrome gene (cytb, 1140 bp) and partial control region (CR, 473 bp) fragments were sequenced using primers L14724 vs. H15915 [28,29] and P vs. E [30], respectively. PCR methods used in this study were the same as those described in Sun et al. [31]. All of the amplified products were purified and sequenced by Shanghai Sangon Biotechnology Co., Ltd. All of the mtDNA sequences of *H. larvatus* were deposited in GenBank with accession numbers MW670581-MW670910 (Supporting Information Table S2). All of the sequences were aligned using GENEIOUS Prime and revised manually.

Seven microsatellite loci (TE2, NEAM15, T121, P6D12, P541, P6C5 and PT5B2) were amplified using fluorescent-labeled primers for 165 individuals. Primer sequences and PCR conditions followed those of Guo et al. [32] and Liu et al. [33]. All of the loci were genotyped using an ABI 3730 automated DNA sequencer with GeneScan 500 LIZ size standard and analyzed by GeneMarker v.2.2.0 (SoftGenetics). For each population, Hardy–Weinberg equilibrium (HWE) and linkage disequilibrium were tested in Genepop v4.3 [34] with 1000 permutations and corrected by Bonferroni correction. All of the loci were screened for null alleles and large allele dropouts using MicroChecker v.2.2.3 [35].

### 3.2. Genetic Analyses

Haplotype diversity (h) and nucleotide diversity (π) were calculated using DnaSP v5 [36] for each population based on mitochondrial cytb and CR. For microsatellites, expected heterozygosity (He) and observed heterozygosity (Ho) were calculated using Genepop; mean allele number (A) and allelic richness (A_R_) per locus were assessed for each population in Fstat 2.9.4 [37].

Tajima’s D [38] estimated in Arlequin v.3.5 [39] was used to test for neutrality. Goodness-of-fit distributions were tested and significant differences from a sudden expansion model assessed using the sum of squared deviations (SSD) and raggedness index (r) with 1000 bootstrap replicates in Arlequin. The time of expansion (t) was estimated from τ = 2ut, where τ is the time to expansion in mutational units and u is the mutation rate per generation for the entire sequence. The u is equal to μgk, where μ is the mutation rate per nucleotide, g is the generation time and k is the sequence length. We used a mutation rate of 1.3 × 10^−8^ per year for cytb based on previous studies on bats [40,41] and a generation time of two years based on data from a congeneric species [21].

The time to the most recent common ancestor (TMRCA) among clades was estimated in BEAST v.2.4.6 [42] based on cytb sequences for those clades with high supports. A strict molecular clock was applied with a fixed mean substitution rate of 1.30 × 10^−8^ subs/site/year. The HKY + I substitution model was used, as determined by Akaike information criterion (AIC) implemented in jModelTest [43]. No outgroup was specified and the constant size was used as a tree prior. An UPGMA tree was used to construct the starting tree. The program was run for 10,000,000 generations (10% discarded as burn-in) and every 1000 was sampled, which were then combined in Tracer v1.6 [44]. ESS values exceeded 2500 for all parameters. All of the other initial parameter settings were the defaults provided by BEAST.

### 3.3. Phylogenetic Analyses

For the mitochondrial analyses, two closely related species, *Hipposideros armiger* (GenBank accession JX849178 and JX014191) and *Hipposideros turpis* (GenBank accession JN247028), were selected as outgroups. Phylogenetic reconstruction using maximum likelihood (ML) in PhyML [45] on T-REX Web Server and Bayesian Inference in MrBayes 3.2.7a [46] on CIPRES was undertaken for the concatenated cytb and CR sequences. We used PartitionFinder2 and the AIC to select the best model of evolution (HKY + I + G for both cytb and CR fragments) for the concatenated mtDNA data for ML and BI analyses. For ML analysis, the starting tree was obtained with BioNJ and the support of the resulting topologies was evaluated using 1000 nonparametric bootstraps. For BI analysis, we used the molecular evolution model parameters estimated for each dataset and two simultaneous runs of Markov chain Monte Carlo (MCMC) analysis, each comprising four chains and 10,000,000 generations. Trees and parameters were sampled every 100 generations. When the run terminated, the deviation of split frequencies reached a value <0.01. The ln-likelihoods of trees reached an asymptote. The first 25% of the sampled trees were discarded as a burn-in. We used PopART [47] to construct a Median-Joining haplotype network for comparison of genealogical relationships among the haplotypes of the concatenated cytb and CR sequences.

For the microsatellite analyses, we used a Bayesian approach by Structure v2.3.4 (Pritchard et al. 2000) using 500,000 iterations after a 100,000-iteration burn-in. The admixture model without a priori designation for populations was used and the number of tested clusters (K) was based on the numbers of populations. For K = 1 to 18, 10 runs were performed for each K value. A structure harvester [48] was used to determine the most likely number of clusters using the ΔK method described by Evanno et al. [49].

### 3.4. Tests for IBD, IBA and IBC

First, we used simple and partial Mantel tests, one of the most widely used approaches to assess spatial processes that drive population structure, to test the correlations between different matrices. Four matrices were built to describe differences between *H. larvatus* populations: (i) mitochondrial genetic distances (given by Fst/(1 − Fst)) between populations were assessed based on the concatenated cytb and CR sequences, with the F_ST_ values calculated by Dnasp; (ii) nuclear genetic distances between populations were calculated using Slatkin’s linearized Fst (given by Fst/(1 − Fst)) based on microsatellites; (iii) geographic distance from the latitude and longitude of each sampling site was calculated by Geographic Distance Matrix Generator online tool (http://biodiversityinformatics.amnh.org/open_source/gdmg); and (iv) climate differences between populations were calculated as the Euclidian distance based on the first three principal components from the PCA using 19 climatic variables. 

Genetic divergence was compared to climate difference and geographic distance using simple Mantel tests, with significance determined by 999 permutation tests. To test for IBD directly, we assessed the correlation between genetic and geographic differences when controlling for climate difference by partial Mantel tests. To test for IBA directly, we assessed correlation between climate and genetic differences when controlling for geographic distance. In addition, we selected mtDNA representing isolation by colonization from glacial refugia and assessed the correlation between nuclear genetic differences and mitochondrial differences when controlling for geographic and climatic differences to test for IBC directly. All of the simple and partial Mantel tests were implemented using the R package “vegan”, with significance determined via 999 permutation tests. To control for multiple tests, *p* values were corrected by the Benjamini and Hochberg [50] criteria using the R package “fdrtool”. 

Second, a redundancy analysis (RDA) was performed to partition the among-population genetic variation into three components, IBD, IBA and IBC. RDA as a multiple linear regression method performed between a matrix of dependent variables and matrices of independent variables is more appropriate than Mantel tests because RDA does not require distance-based metrics and can violate the underlying assumptions of the Mantel test. The dependent matrix contained population allele frequencies of microsatellites of *H. larvatus*. We performed PCA on the allele frequency matrix. PCs 1–10, which cumulatively explained 87.6% of the variation in the microsatellites, were retained for RDA. Three independent matrices were included: (i) the first three principal components (PCs 1–3) of climatic variables (representing IBA), (ii) geographic variables (IBD), and (iii) mtDNA variables (representing isolation by colonization from glacial refugia, IBC). For the geography matrix, we calculated second-order polynomials and combinations of the coordinates of sampling locations (*x*, *y*, *xy*, *x*^2^, *y*^2^) to ensure linear gradients in the data [15]. For the mtDNA matrix, we first estimated the frequencies of each mitotype in each population and then performed PCA on the mitotype frequency matrix. PCs 1–9, which cumulatively explained 78.15% of the variation in mtDNA, were retained for RDA. Among-population variation was partitioned into exclusive effects of climate, geography, isolation by colonization (constrained by the effects of the remaining two independent matrices) and all possible combinations of these three matrices using the varpart and rda functions of the vegan package in R [27]. Significance was tested with the anova.cca function of vegan with a step size of 1000, resulting in at least 999 permutations.

## 4. Results

### 4.1. Mitochondrial Genetic Analyses

For 165 individuals in *H. larvatus* sampled from 18 localities across the entire Chinese range of the species, we examined genetic variation in cytb (1140 bp) and the control region (473 bp). Totals of 53 and 72 haplotypes of cytb and CR were defined, respectively. The alignment of the concatenated cytb and CR (1613 bp) resulted in 94 haplotypes. For cytb, haplotype diversity (h) of each population ranged from 0 (JX and YN2) to 0.956 (HN5) and the nucleotide diversity (π) ranged from 0 (YN2) to 0.01649 (YN1) (Table 1). For CR, h ranged from 0 (JX) to 1 (HN1 and YN1) and π ranged from 0 (GZ) to 0.03284 (YN1) (Table 1).

**Figure 2:**
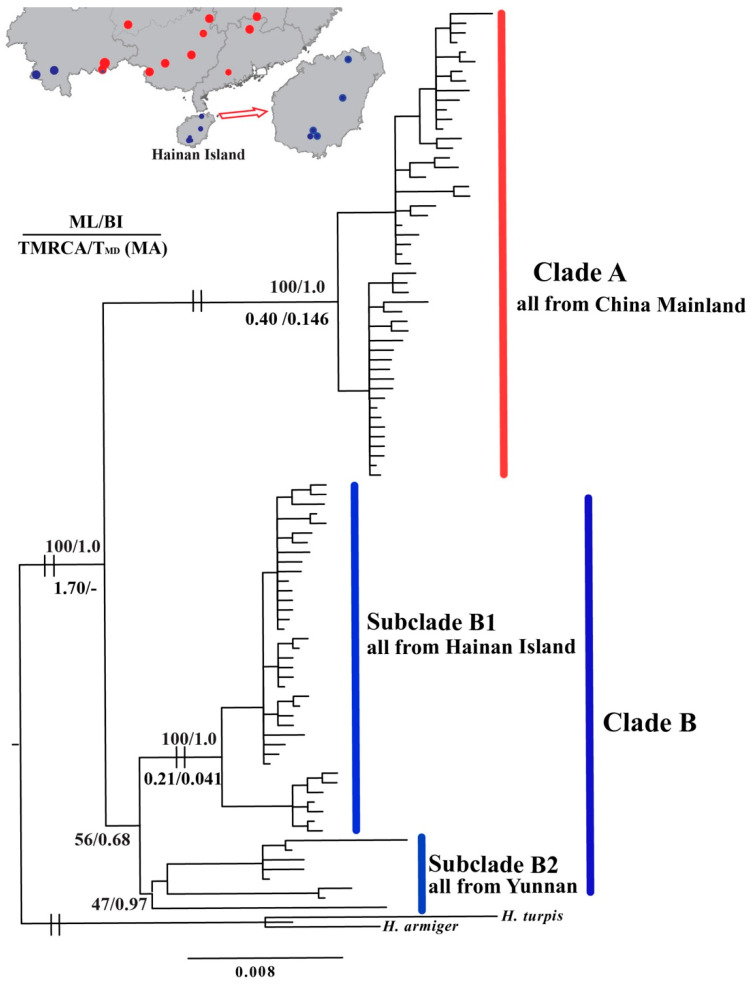
Bayesian phylogenetic tree generated from concatenated mitochondrial cytb and control region sequences of *Hipposideros larvatus*. Values above branches indicate Bayesian posterior probabilities and maximum likelihood bootstrap support values; numbers below branches with high supports are times to the most recent common ancestor estimated using BEAST and estimation of the time of population expansion using mismatch distribution analysis based on a complete cytb gene mutation rate of 0.013 substitutions. The colors are consistent with Figure 1.

All of the individuals formed a highly supported monophyletic lineage (ML: 100%; BI: 1.0) and two clades were recovered by BI and ML phylogenetic reconstruction based on the concatenated mtDNA data (Figure 2). Clade A contained most of the populations from mainland China (ML: 100%; BI: 1.0), while Clade B contained all of the individuals from Hainan Island, named subclade B1 (ML: 100%; BI: 1.0), and some from Yunnan Province (YN2, YN4 and YN1), named subclade B2 (ML: 47%; BI: 0.97) (Figure 2), consistent with the haplotype network (Figure 1b). The ranges of the two clades were almost completely different, while only the YN1 population contained individuals from both Clades A and B (Figure 1a).

For all of the highly supported clades, all *H. larvatus*, Clade A and Clade B1 (Figure 2), the inferred TMRCAs were 1.70 Ma (million years ago) (95% CI: 1.30–2.14 Ma), 0.40 (95% CI: 0.24–0.59 Ma) and 0.21 Ma (95% CI: 0.11–0.33 Ma), respectively (Figure 2). Tajima’s D values indicated that none of the clades or subclades deviated significantly from neutrality (Table S3). The analyses of Clade A and subclade B1 failed to reject the model of population expansion, with estimated timings of expansion occurring 0.146 Ma and 0.041 Ma, respectively (Figure 2, Table S4).

### 4.2. Microsatellite Genetic Analyses

Except for the T121 locus, all of the other six microsatellite loci were polymorphic, with only 4.6% of 108 population-loci combinations significantly deviating from HWE. No sign of linkage disequilibrium was detected. A microchecker identified a potential of 3.8% null alleles in population locus tests. However, null alleles and deviations from HWE were not shown a consistent pattern with any particular locus or population. Thus, all of the loci were retained for subsequent analyses like other studies [31,51]. The number of alleles of each locus ranged from 3.67 (YN2) to 11 (HN3), allelic richness of each locus from 3.667 (YN2) to 5.318 (HN3), Ho from 0.500 (GD2) to 0.845 (JX), and He from 0.708 (YN2) to 0.867 (HN3) (Table 2).

**Figure 3:**
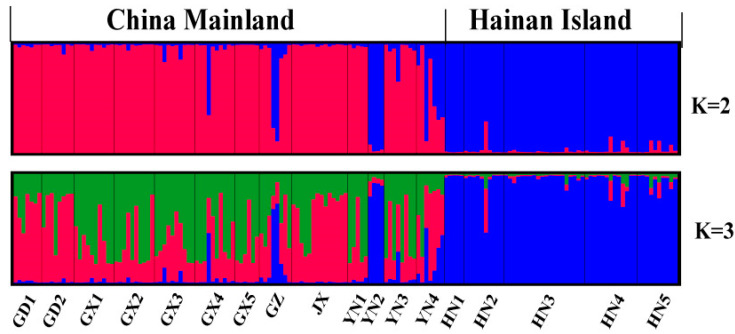
STRUCTURE analysis of *Hipposideros larvatus* nuclear microsatellite genotypes for K = 2 and 3 clusters. Black lines separate individuals of different populations. Population names are labeled under the figure, with their regional affiliations (mainland China and Hainan Island) above it.

Using the Evanno criterion with structure output identified K = 2 as most likely, with the populations from Hainan Island and most populations from mainland China identified as distinct genetic groups (Figure 3). For K = 3 to 18, no new groups were detected. 

### 4.3. Tests for IBD, IBA and IBC

According to the results of simple and partial Mantel tests, there was an overall significant relationship between genetic and geographic distance matrices (*p* < 0.01; Table 2, Figure S2), even when climatic distance was controlled for (all *p* < 0.01, Table 2), suggesting a pattern of isolation by distance. Both nuclear and mitochondrial genetic distances were significantly related to climatic distance (*p* < 0.01; Table 2, Figure S2), even when geographic distance was controlled for (*p* < 0.01; Table 2), suggesting a pattern of isolation by adaptation. Interestingly, we observed that nuclear genetic distance was strongly correlated with mitochondrial distance (r = 0.581, *p* < 0.01; Table 2 and Figure S2), and this relationship remained highly significant after controlling any other variables (*p* < 0.01; Table 2), suggesting a pattern of isolation by colonization.

**Figure 4:**
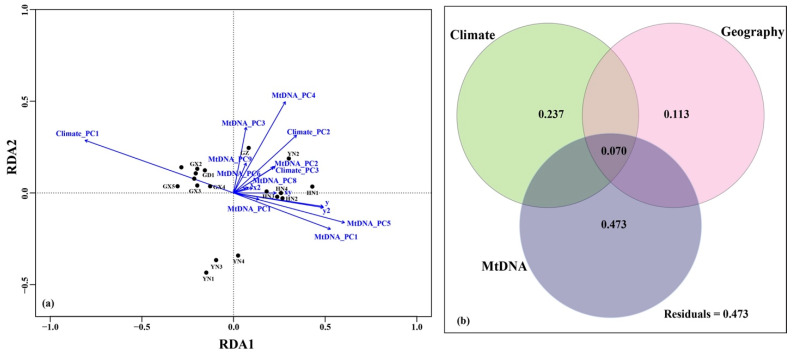
RDA ordination plots (**a**) and a Venn diagram (**b**) of variation partitioning of nuclear divergence in *Hipposideros larvatus*. Nuclear microsatellites, allele frequency matrices, including 10 principal components (NuDNA_PCs 1–10); climate, climatic distance matrices, including three principal components (Climate_PCs 1–3); geography, spatial distance matrices, including five variables (*x*, *y*, *xy*, *x*^2^, *y*^2^); MtDNA, mitotype frequency matrices, including nine principal components (MtDNA_PCs 1–9). The explained variation in variation partitioning is based on adjusted R^2^. Values lower than 0 are not shown.

Redundancy analysis (RDA) suggested that different variables contributing to the nuclear divergence were variable (Figure 4a). RDA axis 1 was most related negatively to the environmental variable (PC1) and positively to the mitochondrial variables (PC1 and PC5) and the geographic variable (y and y^2^). RDA axis 2 was most related positively to the mitochondrial variable (PC3 and PC4) and the climatic variable (PC2) (Figure 4a). The results of variation partitioning analysis also suggested that the mitochondrial variables (R_adj_^2^ = 0.543, *p* = 0.004) and climatic variables (R_adj_^2^ = 0.307, *p* = 0.001) significantly explained the greatest fraction of the nuclear divergence, however, geographic variation explained a smaller fraction with no significant pure effects on nuclear genetic divergence (R_adj_^2^ = 0.183, *p* = 0.186) (Figure 4b).

## 5. Discussion

### 5.1. Phylogeographic History of H. Larvatus

In this study, we investigated the patterns of genetic variation of *H. larvatus* in mainland China and on Hainan Island. We found divergent lineages and a strong genetic structure for nuclear and mitochondrial markers between Hainan Island and mainland China in this bat species (Figure 1, Figure 2 and Figure 3). The genetic discontinuity between Hainan island and mainland China was reported for several other flying organisms, such as the silver pheasant *Lophura nycthemera* [52], the Aisan honey bee *Apis cerana* [22] and several bat species, including *Hipposideros armiger* [21], *Rhinolophus sinicus* [53] and *Pipistrellus abramus* [54]. These are likely related to Pleistocene climatic oscillation and environmental changes.

Our demographic simulations suggested the TMRCA of the entire group of *H. larvatus* populations could be traced back to 1.70 Ma (95% CI: 1.30–2.14 Ma) (Figure 2). During that period, China was experiencing the Poyang glacial stage (1.8 Ma) [55]. Climatic changes and temperature decline may have forced *H. larvatus* to diverge from other bat species and evolve separately in China. Subsequently, different clades were formed in *H. larvatus* in China, including Clade A, containing most of the populations of *H. larvatus* from mainland China, and Clade B, containing all the populations from Hainan Island (subclade B1) and some individuals from Yunnan Province (subclade B2) (Figure 1b and Figure 2). For the highly supported Clade A, the TMRCA was dated to 0.40 Ma (95% CI: 0.24–0.59 Ma), consistent with the Lushan glacial stage (0.4 Ma) [55]. Clade B did not have congruent high supports in ML and BI analyses.

However, interestingly, subclade B1 with all individuals from Hainan Island showed high support (Figure 2), suggesting the potential genetic isolation of Hainan Island. Actually, Hainan Island was repeatedly connected to continental China, when sea levels historically fluctuated and decreased due to Paleoclimate oscillations [56]. The TMRCA of subclade B1 was dated to 0.21 Ma (95% CI: 0.11–0.33 Ma), consistent with the Lulin glacial stage (0.20 Ma) [55] and the Penultimate glaciation (0.33–0.13 Ma) [57]. During 0.30–0.13 Ma, although Hainan Island and the mainland were connected by a land bridge based on isotope analyses of sediment cores [58], sufficient energy may not have been available for long-distance dispersal by bats due to the glacial cold climate.

We also detected a rapid population expansion of subclade B1 in *H. larvatus*, with an estimated time of the expansion occurring 41 Ka (thousand years ago) (95% CI: 15–68 Ka). This was probably driven by the higher precipitation and the warmer climate in China that appeared from 40 to 30 Ka [58]. This postglacial expansion includes the possibility of recent back colonization between Hainan Island and mainland China. Indeed, Hainan Island is separated from the mainland by the Qiongzhou Strait, which averages only 30 km wide, so individual migration could occur via occasional dispersal events, such as extreme winds (typhoons) in the region across the Qiongzhou Strait, just like the genetic admixture in mainland China (Figure 3).

### 5.2. Drives of Genetic Variation in H. Larvatus

Isolation by distance (IBD) is an important factor involved in genetic differentiation among populations [8,59] reported in many species, including birds [60], reptiles [61] and bats [18,21]. In this study, significant positive correlations were observed between nuclear and mitochondrial genetic divergence and geographic distance in *H. larvatus* (Table 2, Figure S2). Interestingly, nuclear divergence showed a higher correlation with geographic distance than mitochondrial divergence both in simple and partial Mantel tests (Table 2). This might be related to sex-biased dispersal in bats [62,63]. Male migrants may have a greater number of long-distance movements and exchange genes over larger geographic scales than philopatric female migrants in *H. larvatus*, leading to the relatively high correlation between nuclear divergence and geographic distance.

Isolation by adaptation (IBA) is another important factor that drives genetic divergence among populations [1]. IBA was detected with limited microsatellite loci in species such as the great snipe [64], moor frog [65], house sparrow [66] and brown booby [67]. Under this pattern, gene flow between populations from ecologically divergent habitats was reduced and genetic differentiation at neutral loci increased along with divergence at loci under selection [1]. Our results suggest that environmental adaptation is a primary driver of the patterns of neutral genetic structure. A significant correlation was observed between genetic divergence and climatic condition (Table 2, Figure S2), even when geographic distance was controlled for (Table 2). However, the results of RDA and variation partitioning also suggested that climatic variables could drive the nuclear divergence based on the significant and relatively high pure effects on nuclear DNA (Figure 4). In addition, obvious genetic differentiation was detected between Hainan Island and the continent (Figure 3). Indeed, different climatic zones occur across mainland Southern China (subtropical monsoon) and Hainan Island (tropical monsoon). The tropical monsoon climate with its high temperature and humidity could provide a favorable environment such as abundant food (insects) for those bats restricted to Hainan Island due to glacial isolation. This environmental adaptation suggests why the Qiongzhou Strait seems to be a major obstacle to migration due to limited individual admixture between the continent and the island. However, the strait averages only 30 km wide and is generally not an obstacle for flying bats (Figure 3).

The isolation by colonization (IBC) pattern is more complex than the IBD and IBA patterns and it is very difficult to dissect IBC from IBD and IBA [1]. However, it was tested in some taxa, such as Berthelot’s pipit (*Anthus berthelotii*) [2] and the waterflea (*Daphnia magna*) [68]. We obtained results suggesting that colonization events contributed to the pattern of neutral genetic structure in *H. larvatus*. Here, we utilized the information from maternally inherited mtDNA as a proxy for colonization, similar to studies on plants such as *Pinus tabuliformis* [15] and *Ficus hirta* [69]. Significant correlations were observed between nuclear divergence and mitochondrial divergence, even when controlling for geographic and environmental distances (Table 2). The results of RDA and variation partitioning suggested that mitochondrial variables (R_adj_^2^ = 0.568, *p* = 0.004) play more important roles in nuclear divergence than geography and the environment (Figure 4). Indeed, the range contractions and expansions of *H. larvatus* driven by climatic oscillations were observed across mainland China and Hainan Island in this study (Table S4), which might explain the contrasting roles of IBC in *H. larvatus*.

## 6. Conclusions

In conclusion, we studied the genetic diversity and evolutionary history of *H. larvatus* across mainland China and Hainan Island. Incorporation of microsatellites, mtDNA, geography and climate data in Mantel tests and RDA analyses offered a better understanding of the complex interactions of IBD, IBA and IBC, as well as their impacts on population genetic structure. The results showed that the observed genetic structure in *H. larvatus* is affected by IBD, IBA and IBC. The combined effects among multiple drivers contribute to the genetic variation and complex evolutionary processes in *H. larvatus*. Furthermore, the genetic differentiation between mainland and the island suggests that it is necessary to integrate the phenotypic, environmental and genetic data to assess the cryptic taxa in *H. larvatus* in further research, like some successful cases [70,71,72]. At the same time, the conservation of island ecosystems coastal areas is of fundamental importance to preserve bat populations on Hainan Island. 

## Figures and Tables

**Table 1:** Sampled populations with their abbreviations and the number (N) of *Hipposideros larvatus* samples used in this study and genetic diversity, including haplotype diversity (h) and nucleotide diversity (π) of the mitochondrial cytb and control region (CR) fragments, and mean number of alleles at each locus (A); allelic richness of each locus (A_R_), observed (Ho) and expected heterozygosity (He) of six microsatellite loci.

Abbrev.	Locality	N	cytb			CR			nSSR			
			Hap	h	π (%)	Hap	h	π (%)	A	A_R_	Ho	He
GD1	Yangchun, Guangdong Province	7	2	0.286	0.025	4	0.714	0.242	6.83	4.972	0.643	0.811
GD2	Shaoguan, Guangdong Province	8	2	0.429	0.038	5	0.893	0.325	6.50	4.701	0.500	0.821
GX1	Congzuo, Guangxi Province	10	5	0.800	0.135	3	0.600	0.156	9	5.174	0.783	0.825
GX2	Longzhou, Guangxi Province	10	5	0.667	0.244	6	0.844	0.753	8.17	5.091	0.717	0.827
GX3	Guilin, Guangxi Province	10	7	0.867	0.17	7	0.911	0.452	7.33	4.634	0.767	0.812
GX4	Laibin, Guangxi Province	10	3	0.378	0.228	4	0.711	0.683	8.5	5.271	0.800	0.860
GX5	Qiepu, Guangxi Province	6	3	0.733	0.076	3	0.733	0.184	4.83	3.977	0.556	0.720
GZ	Lipu, Guizhou Province	8	2	0.250	0.088	3	0.464	0.106	7	4.619	0.688	0.783
JX	Gaozhou, Jiangxi Province	14	1	0.000	0.000	1	0.000	0.000	9.67	5.139	0.845	0.822
YN1	Hekou 1, Yunnan Province	5	4	0.900	1.649	5	1.000	3.284	5.83	5.152	0.700	0.796
YN2	Simao, Yunnan Province	4	1	0.000	0.000	2	0.500	0.317	3.67	3.667	0.708	0.708
YN3	Hekou 2, Yunnan Province	8	3	0.464	0.066	6	0.893	0.409	8.5	5.38	0.625	0.799
YN4	Puer, Yunnan Province	7	5	0.857	0.685	5	0.857	1.271	7	5.059	0.714	0.826
HN1	Baoting 1, Hainan Province	5	3	0.700	0.105	5	1.000	0.508	4.83	4.396	0.533	0.785
HN2	Baoting 2, Hainan Province	10	4	0.733	0.749	7	0.933	0.546	8.33	5.093	0.717	0.840
HN3	Baoting 3, Hainan Province	20	9	0.753	0.119	9	0.847	0.452	11	5.318	0.683	0.867
HN4	Haikou, Hainan Province	13	6	0.821	0.315	8	0.885	1.249	8.5	4.667	0.679	0.809
HN5	Tuncang, Hainan Province	10	8	0.956	0.242	7	0.867	1.045	8.67	5.201	0.750	0.852

**Table 2:** Correlations between microsatellite (Genetic_ssr_) and mitochondrial (Genetic_mt_), climatic (from principal component analysis PCs 1–3) and geographic (km) differences among 18 populations of *Hipposideros larvatus,* tested with simple and partial Mantel tests. Boldface indicates significant *p* values (*p* < 0.05) after multiple testing corrections.

Comparison	r	*p*
**Simple Mantel Tests**		
Genetic_ssr_, geography	0.544	**0.001**
Genetic_ssr_, climate	0.419	**0.001**
Genetic_mt_, geography	0.415	**0.002**
Genetic_mt_, climate	0.445	**0.001**
Genetic_ssr_, genetic_mt_	0.518	**0.001**
**Partial Mantel Tests**		
Genetic_ssr_, geography|climate	0.449	**0.001**
Genetic_ssr_, climate|geography	0.254	**0.008**
Genetic_mt_, geography|climate	0.284	**0.021**
Genetic_mt_, climate|geography	0.330	**0.001**
Genetic_ssr_, genetic_mt_|geography	0.383	**0.001**
Genetic_ssr_, genetic_mt_|climate	0.408	**0.001**

## Data Availability

All relevant data are within the article and its additional files. The sequences data were submitted to the GenBank database under the accession numbers MW670581-MW670910.

## Supplementary Materials

The following are available online at https://www.mdpi.com/2076-2615/11/3/733/s1, Figure S1: Principal component analysis (PCA) of *Hipposideros larvatus*, based on 19 bioclimatic variables across 18 localities. The first two principal components accounted for 54.6% and 20.9% of the variation, respectively. Black dots represent the sampling localities. The code of each bioclimatic variable follows those of WorldClim and ANUCLIM, and is shown in Table S1. Figure S2: Scatter plots of the relationships between genetic distance (F_st_ /1 − F_st_) and geographic distance (**a**,**b**), and climatic distance (**c**,**d**), and between nuclear distance and mitochondrial distance (**e**). F_st_ values were calculated based on nuclear microsatellites (**a**,**c**) and concatenated mitochondrial cytb and control region (**b**,**d**). Correlation coefficient *r* and significance were estimated by Mantel tests. Table S1: The codes of 19 bioclimatic variables used in this study. This scheme follows those of WorldClim and ANUCLIM. Table S2: Sampled populations with geographical coordinates and the GenBank accession numbers of cytb gene and control region sequences of *Hipposideros larvatus.* Table S3: Results of Tajima’s D test for *Hipposideros larvatus* based on the sequences of cytb and CR. None of these appear to be significant. Table S4: Results of mismatch distribution analysis and estimation of the time of population expansion (T_MD_, Ma) for *Hipposideros larvatus* based on the cytb gene. Statistically significant results are indicated by asterisks: * *p* < 0.05, ** *p* < 0.01.
