# Supplementary material for: Effects of Colonization, Geography and Environment on Genetic Divergence in the Intermediate Leaf-Nosed Bat, Hipposideros larvatus
Source: Animals (Basel). 2021 Mar 8;11(3):733. doi: 10.3390/ani11030733 (PMC7998825; doi:10.3390/ani11030733)
Supplement: Supplementary file 1 [file animals-11-00733-s001.pdf]

Effects of colonization, geography and environment on genetic divergence in the intermediate leaf-nosed bat, *Hipposideros larvatus*

Xiangfeng Meng[1], Tong Liu[1], Lin Zhang[1], Tinglei Jiang, Longru Jin[1,*], Keping Sun[1,2,*], Jiang Feng[1,3]

[1] Jilin Provincial Key Laboratory of Animal Resource Conservation and Utilization, Northeast Normal University, Changchun, 130117, China.

[2] Key Laboratory of Vegetation Ecology, Ministry of Education, Changchun, 130024, China.

[3] School of Life Science, Jilin Agricultural University, Changchun, 130118, China.

[*] Authors to whom correspondence should be addressed. E-mail: sunkp129@nenu.edu.cn and jinlr915@nenu.edu.cn

Table S1. The codes of 19 bioclimatic variables used in this study. This scheme follows those of WorldClim and ANUCLIM.

Abbreviation	Bioclimatic Variable
Bio1	Annual Mean Temperature [°C]
Bio2	Mean Diurnal Range [°C]
Bio3	Isothermality (Bio2/Bio7) (* 100)
Bio4	Temperature Seasonality (standard deviation *100)
Bio5	Max Temperature of Warmest Month [°C*10]
Bio6	Min Temperature of Coldest Month [°C*10]
Bio7	Temperature Annual Range (Bio5–Bio6)
Bio8	Mean Temperature of Wettest Quarter [°C*10]
Bio9	Mean Temperature of Driest Quarter [°C*10]
Bio10	Mean Temperature of Warmest Quarter [°C*10]
Bio11	Mean Temperature of Coldest Quarter [°C*10]
Bio12	Annual Precipitation [mm/year]
Bio13	Precipitation of Wettest Month [mm/month]
Bio14	Precipitation of Driest Month [mm/month]
Bio15	Precipitation Seasonality [coefficient of variation]
Bio16	Precipitation of Wettest Quarter [mm/quarter]
Bio17	Precipitation of Driest Quarter [mm/quarter]
Bio18	Precipitation of Warmest Quarter [mm/quarter]
Bio19	Precipitation of Coldest Quarter [mm/quarter]

Table S2. Sampled populations with geographical coordinates and the GenBank accession numbers of cytb gene and control region sequences of *Hipposideros larvatus*.

**Population**	**Longitude**	**Latitude**	**cytb**	**CR**
**GD1**	111.944	22.434	MW670581-MW670586	MW670746-MW670751
**GD2**	113.561	24.772	MW670587-MW670595	MW670752-MW670760
**GX1**	107.824	22.862	MW670596-MW670605	MW670761-MW670770
**GX2**	106.919	22.563	MW670606-MW670615	MW670771-MW670780
**GX3**	110.683	25.413	MW670616-MW670625	MW670781-MW670790
**GX4**	109.674	23.477	MW670626-MW670635	MW670791-MW670800
**GX5**	110.380	24.510	MW670636-MW670641	MW670801-MW670806
**GZ**	105.533	25.283	MW670642-MW670649	MW670807-MW670814
**JX**	114.091	25.462	MW670708-MW670721	MW670873-MW670886
**YN1**	103.847	22.603	MW670722-MW670726	MW670887-MW670891
**YN2**	100.709	22.605	MW670727-MW670730	MW670892-MW670895
**YN3**	103.906	22.743	MW670731-MW670738	MW670896-MW670903
**YN4**	99.550	22.320	MW670739-MW670745	MW670904-MW670910
**HN1**	109.467	18.623	MW670650-MW670654	MW670815-MW670819
**HN2**	109.428	18.598	MW670655-MW670664	MW670820-MW670829
**HN3**	109.448	18.585	MW670665-MW670674<br>MW670688-MW670674	MW670830-MW670839<br>MW670853-MW670862
**HN4**	110.212	19.945	MW670675-MW670687	MW670840-MW670852
**HN5**	110.127	19.231	MW670698-MW670707	MW670863-MW670872

Table S3. Results of Tajima's D test for *Hipposideros larvatus* based on the sequences of cytb and CR. None of these appear to be significant.

	**Tajima's D**
**Cytb**	
**Clade A**	-1.281
**Clade B**	-0.890
**Subclade B1**	-1.298
**Subclade B2**	-0.878
**CR**	
**Clade A**	-1.727
**Clade B**	-1.490
**Subclade B1**	-1.450
**Subclade B2**	-0.362

Table S4. Results of mismatch distribution analysis and estimation of the time of population expansion (T_{MD}, Ma) for *Hipposideros larvatus* based on the cytb gene. Statistically significant results are indicated by asterisks: *P < 0.05, **P < 0.01.

	**SSD**	**r**	**Tao (95% CI)**	**T_{MD} (95% CI)**
**Clade A**	0.031	0.029	8.641(0.479–14.344)	0.146 (0.008–0.237)
**Clade B**				
**Subclade B1**	0.005	0.041	2.438(0.893–4.047)	0.041(0.015–0.068)
**Subclade B2**	0.123*	0.269**	-	-

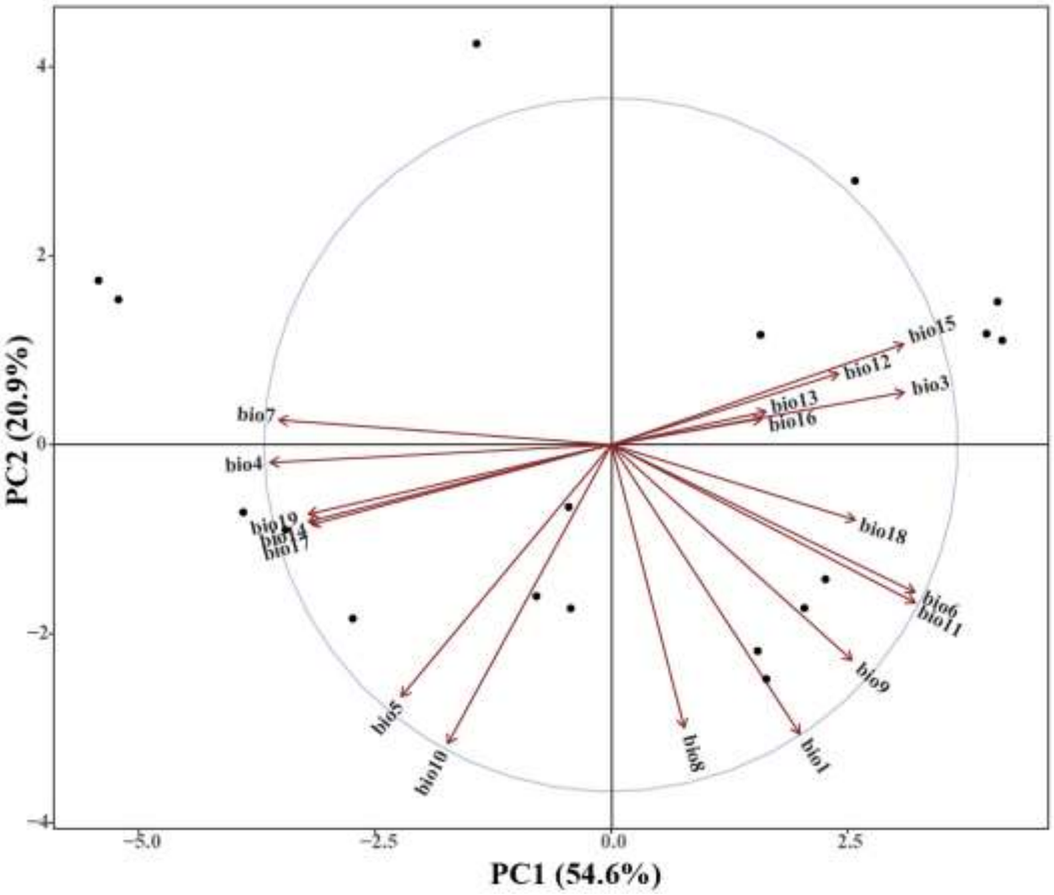

Figure S1. Principle component analysis (PCA) of *Hipposideros larvatus*, based on 19 bioclimatic variables across 18 localities. The first two principle components accounted for 54.6% and 20.9% of the variation, respectively. Black dots represent the sampling localities. The code of each bioclimatic variable follows those of WorldClim and ANUCLIM, and is shown in Table S1.

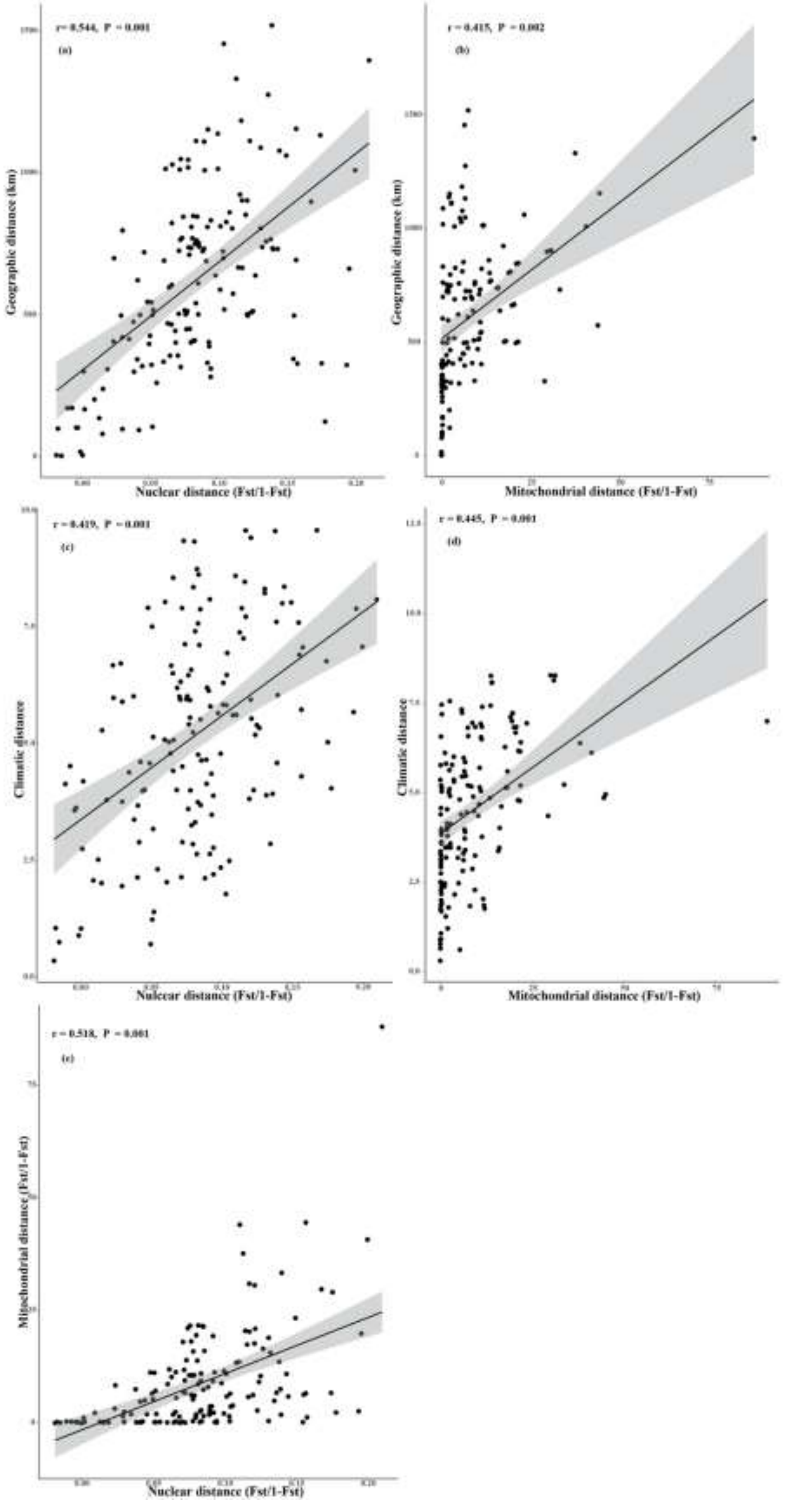
r = 0.544, P = 0.001
(a)
Geographic distance (km)
Nuclear distance (Fst/1-Fst)
r = 0.415, P = 0.002
(b)
Geographic distance (km)
Mitochondrial distance (Fst/1-Fst)
r = 0.419, P = 0.001
(c)
Climatic distance
Nuclear distance (Fst/1-Fst)
r = 0.445, P = 0.001
(d)
Climatic distance
Mitochondrial distance (Fst/1-Fst)
r = 0.518, P = 0.001
(e)
Mitochondrial distance (Fst/1-Fst)
Nuclear distance (Fst/1-Fst)

Figure S2. Scatter plots of the relationships between genetic distance (F_{st} /1- F_{st}) and geographic distance (a, b), and climatic distance (c, d), and between nuclear distance and mitochondrial distance (e). F_{st} values were calculated based on nuclear microsatellites (a, c) and concatenated mitochondrial cytb and control region (b, d). Correlation coefficient *r* and significance were estimated by Mantel tests.
